# Complete Genome Sequence of Flavonifractor plautii JCM 32125^T^

**DOI:** 10.1128/MRA.00135-20

**Published:** 2020-04-23

**Authors:** Dieter M. Tourlousse, Mitsuo Sakamoto, Takamasa Miura, Koji Narita, Akiko Ohashi, Yoshihito Uchino, Atsushi Yamazoe, Keishi Kameyama, Jun Terauchi, Moriya Ohkuma, Hiroko Kawasaki, Yuji Sekiguchi

**Affiliations:** a Biomedical Research Institute, National Institute of Advanced Industrial Science and Technology (AIST), Tsukuba, Ibaraki, Japan; b Microbe Division/Japan Collection of Microorganisms, RIKEN BioResource Research Center, Tsukuba, Ibaraki, Japan; c Biological Resource Center, National Institute of Technology and Evaluation (NITE), Kisarazu, Chiba, Japan; d Japan Microbiome Consortium (JMBC), Osaka, Osaka, Japan; University of Maryland School of Medicine

## Abstract

We report the complete genome sequence of Flavonifractor plautii JCM 32125^T^ (=VPI 0310^T^). The genome consists of a single circular chromosome of 3,985,392 bp (G+C content, 60.9%) and was predicted to contain 3 complete sets of rRNA genes, 63 tRNA genes, and 3,764 protein-coding sequences.

## ANNOUNCEMENT

Flavonifractor plautii (formerly Eubacterium plautii) is a butyrate producer that is highly prevalent in the human gastrointestinal tract (1). The species is well known for its ability to convert a wide range of dietary flavonoids (2), thereby playing a role in shaping gut ecology and influencing host health. The abundance of *F. plautii* was recently found to be elevated in colorectal cancer patients in India, which was presumed to be associated with the conversion of anticarcinogenic flavonoids by *F. plautii* (3). In Japan, *F. plautii* was shown to be more abundant in nonobese individuals than in obese persons (4). In this work, we generated a complete genome sequence of the authentic type strain of *F. plautii* (JCM 32125^T^ = VPI 0310^T^ [5]) using Illumina and Oxford Nanopore Technologies (ONT) sequencing.

Cells from the Japan Collection of Microorganisms were cultured under an N_2_ atmosphere in modified GAM broth with 1% glucose, and DNA was purified using the EZ1 DNA tissue kit (Qiagen). Libraries for Illumina sequencing were prepared using the TruSeq Nano DNA kit and sequenced on a MiSeq instrument (2 × 251-bp reads) at a coverage of ∼180×. Libraries for ONT sequencing were generated with the ligation sequencing kit (SQK-LSK109) using the native barcoding expansion pack (EXP-NBD104) for library multiplexing; sequencing was performed on an R9.4.1 flow cell (FLO-MIN106) using the MinION device. All software tools for read processing and assembly were run with default settings unless indicated otherwise. Quality control of Illumina reads was performed using Trimmomatic v0.38 (6); 3,360,253 quality-filtered reads were retained for assembly. For ONT sequencing data, Guppy v3.1.5 (ONT) was used for base calling in high-accuracy mode with demultiplexing and trimming of barcodes; reads with a quality score of <9 and size of <1,000 bp were discarded using NanoFilt v2.5.0 (7). Finally, Filtlong v0.2.0 (https://github.com/rrwick/Filtlong) was used to obtain a subset of high-quality reads by using the Illumina reads as external references and discarding the poorest 10% of the read bases. A total of 53,165 ONT reads (*N*_50_, 10,089 bp; coverage, ∼120×) were used to generate a long-read assembly using Flye v2.5 (8). This assembly was then combined with the Illumina reads to generate a final assembly using Unicycler v0.4.7 (9). Annotation was performed with the NCBI Prokaryotic Genome Annotation Pipeline v4.11 (10).

The genome of *F. plautii* JCM 32125^T^ consists of a single circular chromosome 3,985,392 bp long. The genome has a G+C content of 60.9% and was predicted to contain 3 complete rRNA operons and 63 tRNA genes and encode for 3,764 proteins. We expect that the availability of the complete genome of the type strain of *F. plautii* reported here will provide valuable information for further characterization of the role of this bacterium in the gut, for example, through mining of the genome for genes involved in the conversion of flavonoids.

### Data availability.

This genome sequence has been deposited in DDBJ/EMBL/GenBank under the accession number CP048436. The raw ONT and Illumina sequencing reads are available in the Sequence Read Archive (SRA) under accession numbers SRR10968456 and SRR10968459, respectively.
